# When state Medicaid demonstrations end: projected eligibility loss after a MassHealth housing support program transition

**DOI:** 10.1093/haschl/qxaf213

**Published:** 2025-11-10

**Authors:** Nicole C McCann, Heather E Hsu, Stephanie Ettinger de Cuba, Jasper Frank, Paulina Lange, Michael D Stein, Paul R Shafer

**Affiliations:** Department of Health Law, Policy, and Management, Boston University School of Public Health, Boston, MA 02118, United States; Department of Pediatrics, Boston Medical Center, Boston, MA 02118, United States; Department of Pediatrics, Boston University Chobanian and Avedisian School of Medicine, Boston, MA 02118, United States; Department of Health Law, Policy, and Management, Boston University School of Public Health, Boston, MA 02118, United States; Department of Pediatrics, Boston University Chobanian and Avedisian School of Medicine, Boston, MA 02118, United States; Department of Population Health, Boston Medical Center, Boston, MA 02118, United States; Department of Population Health, Boston Medical Center, Boston, MA 02118, United States; Department of Health Law, Policy, and Management, Boston University School of Public Health, Boston, MA 02118, United States; Department of Health Law, Policy, and Management, Boston University School of Public Health, Boston, MA 02118, United States

**Keywords:** Medicaid, demonstration waivers, accountable care organizations, housing programs, health-related social needs

## Abstract

**Introduction:**

Massachusetts Medicaid (MassHealth) transitioned a Section 1115 waiver-based housing support program to a permanent program in 2025, implementing new eligibility restrictions.

**Methods:**

We projected the potential impact of these new eligibility restrictions using linked administrative claims and programmatic data.

**Results:**

Among individuals enrolled from 2021 to 2024, 68% would no longer qualify under updated 2025 criteria. Those projected to remain eligible were older and had more chronic conditions and higher healthcare utilization than those projected to be ineligible, but similar prevalence of opioid use disorder and mental illness.

**Conclusion:**

These findings highlight the potential for unintended consequences in eligibility prioritization stemming from operational shifts in Medicaid waiver-based programs.

## Introduction

Up to half of variation in health outcomes is attributable to social drivers of health, the conditions in which people live, work, and age.^1^ Given this, health entities, including payors, have increasingly focused on addressing health-related social needs (HRSN), the adverse social conditions, like homelessness or food insecurity, that contribute to poor health outcomes.^1^ Currently, 25 states, including Massachusetts, have approved Medicaid Section 1115 waivers that include demonstration projects targeting HRSN.^2^ Almost all of these projects incorporate housing supports, given rising rates of homelessness and housing instability,^3,4^ and evidence linking housing to improved health outcomes and lower healthcare costs.^5,6^ Demonstration projects are typically approved for 3- to 5-year periods, after which they may be continued, amended, or terminated in subsequent waivers. Few studies have assessed the effects of scaling back HRSN-focused demonstration projects. While the changes evaluated in this study occurred before the Centers for Medicare and Medicaid Services (CMS) rescinded Biden Administration-era HRSN guidance in March 2025,^7^ understanding the effects of program transitions is increasingly important, as similar changes may become more common following this HRSN guidance withdrawal.

In 2020, Massachusetts Medicaid (MassHealth) launched the Flexible Services Program (FSP) under a Section 1115 waiver to provide nutrition and housing supports to Accountable Care Organization (ACO) members, aiming to improve health outcomes and reduce healthcare costs.^8^ Through FSP, ACOs partnered with social service organizations (SSOs) to deliver these supports. To be eligible for FSP for housing supports (FSP-H), members needed at least 1 housing need (ie, homelessness or housing instability), and 1 health need (eg, behavioral health condition or requiring assistance with daily living).^9^ FSP-H services included pretenancy supports (eg, case management, one-time set up costs), tenancy-sustaining supports (eg, tenancy training), and home modifications (eg, mold remediation).^9^

In 2022, Massachusetts received authority to amend the 2020 Section 1115 waiver to integrate FSP services into the ACO managed care system, transitioning HRSN supports from a waiver-based experiment to billable medical services; this change was scheduled to begin January 1, 2025.^10^ At the same time, MassHealth also restricted FSP program eligibility in response to CMS funding constraints and guidance that shifted all financial risk to the state, ensuring service delivery did not exceed available state funds. As of January 2025, the program still requires a housing and health need for eligibility, but pretenancy supports are now limited to members aged 55+ who meet a narrower homelessness definition (eg, excluding those who are doubled up with family/friends), and tenancy-sustaining supports require a lease violation and history of past-year emergency department use. This study describes individuals enrolled in FSP-H from 2021 to 2024 and projects how the 2025 eligibility changes potentially affect enrollment.

## Methods

This study focuses on FSP-H within the WellSense Community Alliance ACO (WCACO), which includes New England's largest safety-net hospital (Boston Medical Center [BMC]) and affiliated federally qualified health centers.^11,12^ BMC Health System maintains internal FSP-H programmatic data, which contains information on WCACO members' program enrollment date, their housing-related need (homelessness or housing instability), the date and type of housing support services received, and unique identifiers for linkage with WellSense claims data which includes MassHealth plans. Individuals were included in our primary analytic sample if they had received at least 1 FSP-H service between March 2021 and November 2024.

Our WellSense claims dataset contained a demographic file as well as statewide encounter-level files with encounter type and diagnostic and procedure codes from 2018 to 2024 for all WCACO members. Demographic variables available included age, sex assigned at birth, race, ethnicity, and preferred language. We created clinical characteristic variables related to FSP-H health need eligibility criteria for the 12-month period leading up to individuals' FSP-H enrollment month, including a modified Elixhauser score (which measures comorbidities, excluding behavioral health conditions that we reported separately^13^), and having at least 1 International Classification of Diseases 10th edition (ICD-10) code for opioid use disorder, alcohol use disorder, any substance use disorder, serious mental illness, any mental illness, or an ICD-10 or procedure code for frailty associated with needing assistance with activities of daily living (ADL) (Table S1). We also calculated individuals' median (interquartile range [IQR]) number of inpatient hospitalizations, ED visits, and outpatient visits over this period.

For our analytic sample, we described the number and types of FSP-H services provided and median length of program enrollment. We also described demographic and clinical characteristics of the sample. To project the potential impact of the 2025 changes to the program, we stratified the sample into 2 groups: (1) individuals expected to maintain eligibility under the new criteria, and (2) individuals expected to lose eligibility. We defined those maintaining eligibility as either (1) individuals enrolled for pretenancy services who met the new age restriction criteria, or (2) individuals enrolled for tenancy-sustaining services who met the new ED utilization criteria. We compared demographic and clinical characteristics between those who would maintain eligibility and those who would no longer be eligible, using χ^2^ tests to test for significant differences at the *P* < 0.05 level.

This analysis has several limitations. We were unable to account for the eligibility criteria changes not documented in health records, including the changed homelessness definition and lease violation requirement. Thus, we likely underestimated the proportion of FSP-H enrollees who would no longer be eligible. Second, our claims-based demographic data has high missingness, limiting our ability to draw conclusions around the potential impact of eligibility criteria changes on racial/ethnic and language-based disparities. Further, nonmeasurable eligibility restrictions (eg, required lease violation) may be nonrandomly distributed across sociodemographic or clinical characteristics, and could contribute to disparities.

## Results

Our analytic sample included 437 WCACO members enrolled in FSP-H from 2021 to 2024. At enrollment, 297 (68%) were experiencing homelessness and received pretenancy services, while 140 (32%) were at risk of homelessness and received tenancy-sustaining services. The median (IQR) number of total services received per member was 2 (1-12) and the median (IQR) number of days of program enrollment was 62 (29-755).

FSP-H enrollees were majority male (53%) with a plurality aged 41-54 years (35%). 27% of enrollees were Black, 19% were white, and 7% were Hispanic, with the remainder missing race/ethnicity data (Table 1). A slight majority (51%) had English recorded as their preferred language, 7% had a non-English preferred language, and the rest had missing language data. Over the 12 months prior to FSP-H enrollment, most enrollees (54%) had 3+ conditions on the modified Elixhauser score, as well as at least 1 ICD-10 code indicating a substance use disorder (53%), serious mental illness (57%), or frailty associated with ADL (77%). The median (IQR) number of past-year hospitalizations, ED visits, and outpatient visits were 1 (0-32), 1 (0-42), and 17 (8-366), respectively.

**Table 1.** Demographic and clinical characteristics of WCACO members who received MassHealth Flexible Services Program housing supports, stratified by projected 2025 program eligibility.

Characteristic	In FSP-H 2021-2024 (*N* = 437), *N* (%)	No longer eligible for housing supports, 2025 (*N* = 295), *N* (%)	Still eligible for housing supports, 2025 (*N* = 142), *N* (%)	*P*-value
Age category^a^				
17-30	46 (11)	41 (14)	<11 (<8)^b^	<0.001^a^
31-54	248 (57)	225 (76)	23 (16)	
55+	143 (33)	29 (10)	>96 (>71)^b^	
Sex assigned at birth				
Male	232 (53)	149 (51)	83 (58)	0.119
Female	205 (47)	146 (49)	59 (42)	
Race/ethnicity^c,d^				
White race	84 (19)	56 (19)	28 (20)	0.892
Black race/Hispanic ethnicity	146 (33)	98 (33)	48 (34)	0.169^d^/0.060^d^
Non-English preferred language^c^	31 (7)	23 (8)	<11 (−)^b^	0.298
Modified Elixhauser score				
No conditions	72 (16)	63 (21)	<11 (<8)^b^	<0.001
1 Condition	69 (16)	53 (18)	16 (11)	
2 Conditions	61 (14)	44 (15)	17 (12)	
3+ Conditions	235 (54)	135 (46)	>85 (>63)^b^	
Opioid use disorder^e^	142 (32)	97 (33)	45 (32)	0.803
Alcohol use disorder^e^	138 (32)	84 (28)	54 (38)	0.044
Any substance use disorder^e^	231 (53)	147 (50)	84 (59)	0.067
Serious mental illness^e^	249 (57)	164 (56)	85 (60)	0.399
Any mental illness^e^	310 (71)	204 (69)	106 (75)	0.236
Frailty (ADL difficulty)^e^	338 (77)	212 (72)	126 (89)	<0.001
Median past-year (IQR) hospitalizations	1 (0-32)	1 (0-22)	2 (0-29)	<0.001
Median past-year (IQR) ED visits	1 (0-42)	1 (0-18)	3 (1-34)	<0.001
Median past-year (IQR) outpatient visits	17 (8-366)	16 (7-313)	19 (10-302)	0.060

Abbreviations: ADL, activities of daily living; FSP-H, flexible services program housing supports; IQR, interquartile range; WCACO, WellSense Community Alliance Accountable Care Organization.

^a^Age categories were aggregated to comply with MassHealth guidelines for suppression of cells with counts <11; statistical testing was conducted for the following age categories: 17-21, 22-30, 31-40, 41-54, and 55+ years.

^b^Suppressed due to MassHealth guidelines for cell counts <11 or to prevent back-calculation of small cell counts.

^c^“Other” race/ethnicity categories were too infrequent to present in table (<2%); 46% of participants had missing race/ethnicity data; 42% had missing language data; missing values excluded from statistical testing.

^d^“Black” race and “Hispanic” ethnicity counts are aggregated to comply with MassHealth guidelines for suppression of cell counts <11. However, statistical testing was conducted for Black race and Hispanic ethnicity separately, and respective *P*-values are shown.

^e^At least 1 past-year International Classification of Disease (10th Edition) code, or procedure code; see Supplemental Appendix for codes.

**Figure 1.**
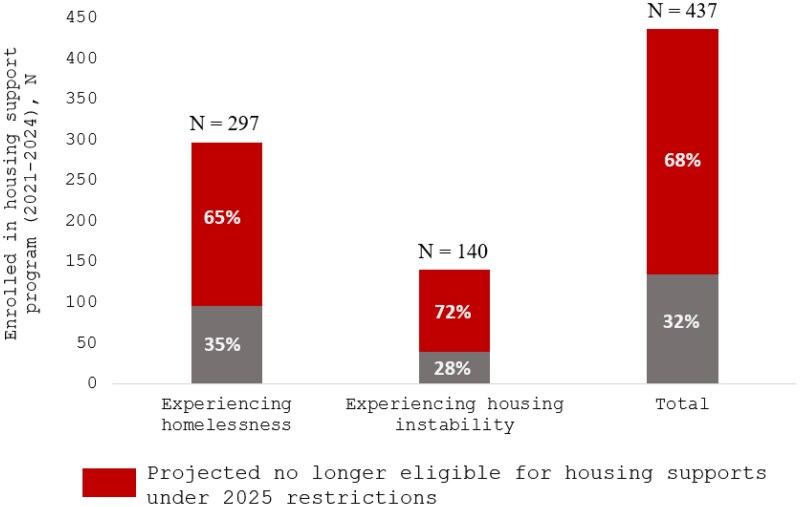
Accountable Care Organization Members enrolled in Massachusetts Medicaid (MassHealth) Flexible Services Program Housing Supports from 2021 to 2024 and projected eligibility loss following 2025 program transition.^1^

We found that 68% of our sample enrolled in FSP-H were projected to no longer be eligible for the new 2025 HRSN Services housing support program. Among those experiencing homelessness, 194/297 (65%) would no longer be eligible due to age, and among those at risk of homelessness, 101/140 (72%) would no longer be eligible because they did not meet the ED utilization history criteria (Figure 1). Those projected to maintain eligibility had a significantly higher prevalence of high Elixhauser scores, frailty, alcohol use disorder, and past-year inpatient and ED utilization than those no longer eligible, and were older (Table 1). There were no significant differences in the other reported demographics or clinical characteristics, including opioid use disorder and mental illnesses, based on projected eligibility retention (Table 1).

## Discussion

We highlight a case where operational changes to a Medicaid waiver-based housing support initiative are projected to restrict patients' eligibility for receiving housing support services through their Medicaid plan. Of those who were previously deemed in need and enrolled in FSP-H, an estimated 70% would no longer be eligible under new criteria. We found that those projected to remain eligible would be significantly older with higher prevalence of certain conditions, like frailty, but similar prevalence of others, including opioid use disorder and mental illness. These prioritizations may unintentionally exclude high-need individuals, with limited evidence justifying the shift.

Although Medicaid demonstration projects are intended to test and refine interventions through evaluation, in practice, robust evaluations are rare,^14^ and waiver-based programs are vulnerable to swings in policy preferences.^15^ In Massachusetts, FSP-H shifted substantially, largely in response to operational changes in CMS funding and guidance, with projected significant effects on not just the number of eligible individuals, but the clinical makeup of the eligible population. The unintended consequences of this are unclear, as there is little available evidence supporting different prioritization schemes in Medicaid-based housing support programs.

Further research to understand the potential impacts of shifts in HRSN-focused waiver programs is especially important given current policy uncertainty, with Biden-era CMS HRSN guidance rescinded and future waiver approvals depending on case-by-case decisions under the Trump Administration.^7^ Rescinding this CMS guidance creates uncertainty for states, which may reduce creation and improvement of cost-effective programs, limiting program scalability.^16^ Already in 2025, a first-in-the-nation Medicaid waiver-based initiative in North Carolina was shuttered.^17^

In the context of declining federal support, building an evidence base on effectiveness and cost-effectiveness of Medicaid housing support programs, their eligibility structures, and the impact of program cuts, is essential for long-term sustainability. High-quality evidence generation is needed so that demonstration projects can function as experiments, as intended, rather than contracting or expanding programs based primarily on operational, administrative, or political conditions.

## Supplementary Material

qxaf213_Supplementary_Data
