# Supplementary material for: When state Medicaid demonstrations end: projected eligibility loss after a MassHealth housing support program transition
Source: Health Aff Sch. 2025 Nov 10;3(11):qxaf213. doi: 10.1093/haschl/qxaf213 (PMC12628790; doi:10.1093/haschl/qxaf213)
Supplement: qxaf213_Supplementary_Data [file qxaf213_supplementary_data.zip › McCann_supplement.docx]

**Supplemental Appendix: When state Medicaid demonstrations end: projected eligibility loss after a MassHealth housing support program transition**

Nicole C. McCann, Heather E. Hsu, Stephanie Ettinger de Cuba, Jasper Frank, Paulina Lange,

Michael D. Stein, Paul R. Shafer

**Table of contents.**

**Supplemental Table.** ICD and procedure codes used to estimate MassHealth housing support program eligibility (page 2)

**Supplemental Table. ICD and procedure codes used to project MassHealth housing support program eligibility**

| **Eligibility-related construct** | **Included conditions** | **Codes** |
| --- | --- | --- |
| Opioid use disorder^1^ | Opioid abuse;  Opioid dependence; Opioid use, unspecified | *ICD-10*:  F111x; F112x, F119x |
| Alcohol use disorder^1^ | Alcohol abuse; alcohol dependence; alcohol use unspecified; alcoholic polyneuropathy; Alcoholic cardiomyopathy; Alcoholic gastritis without bleeding; Alcoholic gastritis with bleeding; Alcoholic hepatitis without ascites; Alcoholic hepatitis with ascites; Alcohol use complicating pregnancy, unspecified trimester; Alcohol use complicating pregnancy, first trimester; Alcohol use complicating pregnancy, second trimester; Alcohol use complicating pregnancy, third trimester; Alcohol use complicating childbirth; Alcohol use complicating the puerperium | *ICD-10*  F1010; F1011; F10120; F10121; F10129; F10130; F10131; F10132; F10139; F1014; F10150; F10151; F10159; F10180; F10181; F10182; F10188; F1019; F1020; F1021; F10220; F10221; F10229; F10230; F10231; F10232; F10239; F1024; F10250; F10251; F10259; F1026; F1027; F10280; F10281; F10282; F10288; F1029; F1094; F10950; F10951; F10959; F1096; F1097; F10980; G621; I426; K2920; K2921; K7010; K7011; O99310; O99311; O99312 |
| Any substance use disorder^1^ | Opioid abuse;  Opioid dependence; Opioid use, unspecified; Alcohol abuse; alcohol dependence; alcohol use unspecified; alcoholic polyneuropathy; Alcoholic cardiomyopathy; Alcoholic gastritis without bleeding; Alcoholic gastritis with bleeding; Alcoholic hepatitis without ascites; Alcoholic hepatitis with ascites; Alcohol use complicating pregnancy, unspecified trimester; Alcohol use complicating pregnancy, first trimester; Alcohol use complicating pregnancy, second trimester; Alcohol use complicating pregnancy, third trimester; Alcohol use complicating childbirth; Alcohol use complicating the puerperium; Cannabis abuse; Cannabis dependence; Sedative, hypnotic, or anxiolytic abuse; Sedative, hypnotic, or anxiolytic dependence; Cocaine abuse; Cocaine dependence; Other stimulant abuse; Other stimulant dependence; Hallucinogen abuse; Hallucinogen dependence; Inhalant abuse; Inhalant dependence; Other psychoactive substance abuse; Other psychoactive substance dependence; Drug use complicating pregnancy, unspecified trimester; Drug use complicating pregnancy, first trimester; Drug use complicating pregnancy, second trimester; Drug use complicating pregnancy, third trimester; Drug use complicating childbirth; Drug use complicating the puerperium | *ICD-10:* F111x; F112x, F119x; F1010; F1011; F10120; F10121; F10129; F10130; F10131; F10132; F10139; F1014; F10150; F10151; F10159; F10180; F10181; F10182; F10188; F1019; F1020; F1021; F10220; F10221; F10229; F10230; F10231; F10232; F10239; F1024; F10250; F10251; F10259; F1026; F1027; F10280; F10281; F10282; F10288; F1029; F1094; F10950; F10951; F10959; F1096; F1097; F10980; G621; I426; K2920; K2921; K7010; K7011; O99310; O99311; O99312; F1210; F1211; F12120; F12121; F12122; F12129; F1213; F12150; F12151; F12159; F12180; F12188; F1219; F1220; F1221; F12220; F12221; F12222; F12229; F1223; F12250; F12251; F12259; F12280; F12288; F1229; F1310; F1311; F13120; F13121; F13129; F13130; F13131; F13132; F13139; F1314; F13150; F13151; F13159; F13180; F13181; F13182; F13188; F1319; F1320; F1321; F13220; F13221; F13229; F13230; F13231; F13232; F13239; F1324; F13250; F13251; F13259; F1326; F1327; F13280; F13281; F13282; F13288; F1329; F1410; F1411; F14120; F14121; F14122; F14129; F1413; F1414; F14150; F14151; F14159; F14180; F14181; F14182; F14188; F1419; F1420; F1421; F14220; F14221; F14222; F14229; F1423; F1424; F14250; F14251; F14259; F14280; F14281; F14282; F14288; F1429; F1510; F1511; F15120; F15121; F15122; F15129; F1513; F1514; F15150; F15151; F15159; F15180; F15181; F15182; F15188; F1519; F1520; F1521; F15220; F15221; F15222; F15229; F1523; F1524; F15250; F15251; F15259; F15280; F15281; F15282; F15288; F1529; F1610; F1611; F16120; F16121; F16122; F16129; F1614; F16150; F16151; F16159; F16180; F16183; F16188; F1619; F1620; F1621; F16220; F16221; F16229; F1624; F16250; F16251; F16259; F16280; F16283; F16288; F1629; F1810; F1811; F18120; F18121; F18129; F1814; F18150; F18151; F18159; F1817; F18180; F18188; F1819; F1820; F1821; F18220; F18221; F18229; F1824; F18250; F18251; F18259; F1827; F18280; F18288; F1829; F1910; F1911; F19120; F19121; F19122; F19129; F19130; F19131; F19132; F19139; F1914; F19150; F19151; F19159; F1916; F1917; F19180; F19181; F19182; F19188; F1919; F1920; F1921; F19220; F19221; F19222; F19229; F19230; F19231; F19232; F19239; F1924; F19250; F19251; F19259; F1926; F1927; F19280; F19281; F19282; F19288; F1929; O99320; O99321; O99322; O99323; O99324; O99325 |
| Serious mental illness^1^ | Major depressive disorder, single episode, severe without psychotic features; Major depressive disorder, single episode, severe with psychotic features; Major depressive disorder, recurrent severe without psychotic features; Major depressive disorder, recurrent, severe with psychotic symptoms; bipolar disorder (excluding cyclothymic disorder); Schizophrenia; Delusional disorders; shared psychotic disorder; Schizoaffective disorder, Other psychotic disorder; Unspecified psychosis | *ICD-10:* F320; F321; F322; F323; F324; F325; F329; F330; F331; F332; F333; F3340; F3341; F3342; F339; F3010; F3011; F3012; F3013; F302; F303; F304; F308; F309; F31x; F200; F201; F202; F203; F205; F2081; F2089; F209; F22; F24; F250; F251; F258; F259; F28; F29 |
| Any mental illness^1^ | Mood disorder; Major depressive disorder; Depression; Depressive episodes; Dysthymic disorder; Anxiety; Attention deficit hyperactivity disorder; Post-traumatic stress disorder; bipolar disorder (including cyclothymic disorder); Schizophrenia; Delusional disorders; psychotic disorder; shared psychotic disorder; Catatonic disorder; Schizoaffective disorder; Other psychotic disorder; Unspecified psychosis; Mood disorder; Brief psychotic disorder; Mental and behavioral disorders associated with puerperium | *ICD-10:* F320; F321; F322; F323; F324; F325; F329; F330; F331; F332; F333; F3340; F3341; F3342; F339; F0631; F0632; F0634; F328; F3289; F32A; F338; F341; F064; F4000; F4001; F4002; F401; F4010; F4011; F402; F40210; F40218; F40220; F40228; F4023; F40230; F40231; F40232; F40233; F4024; F40240; F40241; F40242; F40243; F40248; F4029; F40290; F40298; F408; F409; F41; F410; F411; F413; F418; F419; F930; F940; F90; F900; F901; F902; F908; F909; F430; F4310; F4311; F4312; F438; F439; F941; F942; F3010; F3011; F3012; F3013; F302; F303; F304; F308; F309; F31; F340; F200; F201; F202; F203; F205; F2081; F2089; F209; F22; F24; F250; F251; F258; F259; F28; F29; F060; F061; F062; F063; F23; F25; F53 |
| Frailty code, related to difficulty with Activities of Daily Living^2^ | Lipid abnormality; Vertigo; Arthritis and joint conditions; Bladder disfunction; Podiatric care; Heart failure; Psychiatric care; Hypotension or shock; Stroke or brain injury; Dementias; Parkinson’s; Weakness; Skin ulcer; Paralysis; Rehabilitation care; Home oxygen; Ambulance transport; Wheelchair; Home hospital bed; | *ICD-10*: E71.30; E75.21; E75.22; E75.24; E75.3; E75.5; E75.6; E77.0; E78.0; E78.1; E78.2; E78.3; E78.4; E78.5; E78.6; E78.70; E78.79; E78.8; E78.9; E88.1; E88.89; H81.; H82.; H83.0; H83.1; H83.2; R42.; M00.; M01.; M02.1; M02.3; M02.8; M04.2; M04.8; M04.9; M05.; M06.0; M06.1; M06.3; M06.4; M06.8; M06.9; M07.6; M08.0; M08.2; M08.3; M08.4; M08.8; M08.9; M11.1; M11.2; M11.8; M11.9; M12.0; M12.8; M12.9; M13.0; M13.1; M15.; M16.; M17.; M18.; M19.0; M19.1; M19.2; M19.9; M22.0; M22.1; M23.5; M24.0; M24.1; M24.3; M24.4; M24.5; M24.6; M24.7; M24.8; M24.9; M25.0; M25.2; M25.3; M25.4; M25.5; M25.6; M25.9; M32.10; M32.12; M32.13; M32.14; M32.15; M32.19; M32.8; M32.9; M33.; M34.; M35.00; M35.01; M35.02; M35.03; M35.04; M35.09; M35.1; M35.2; M35.3; M35.5; M35.8; M35.9; M36.8; M43.3; M43.4; M43.5X2; M43.5X3; M43.5X4; M43.5X5; M43.5X6; M43.5X7; M43.5X8; M43.5X9; M79.646; N13.; N31.; N32.; N36.; N39.3; N39.4; N39.8; N39.9; R32.; R33.; R39.14; R39.81; L02.61; L03.03; L03.04; L60.; L62.; L84.; I09.81; I11.0; I13.0; I13.2; I25.5; I42.; I43.; I50.; I51.4; I51.5; I51.7; I97.0; I97.11; I97.120; I97.13; I97.19; F01.; F02.; F03.; F04.; F05.; F06.; F07.; F09.; F10.13; F10.14; F10.15; F10.180; F10.182; F10.188; F10.23; F10.24; F10.25; F10.26; F10.27; F10.280; F10.282; F10.93; F10.94; F10.95; F10.96; F10.97; F10.980; F10.982; F11.13; F11.14; F11.15; F11.182; F11.23; F11.24; F11.25; F11.282; F11.93; F11.94; F11.95; F11.982; F12.13; F12.15; F12.180; F12.23; F12.25; F12.280; F12.93; F12.95; F12.980; F13.13; F13.14; F13.15; F13.180; F13.182; F13.23; F13.24; F13.25; F13.26; F13.27; F13.280; F13.282; F13.93; F13.94; F13.95; F13.96; F13.97; F13.980; F13.982; F14.13; F14.14; F14.15; F14.180; F14.182; F14.23; F14.24; F14.25; F14.280; F14.282; F14.93; F14.94; F14.95; F14.980; F14.982; F15.13; F15.14; F15.15; F15.180; F15.182; F15.23; F15.24; F15.25; F15.280; Z51.89; I95.; R57.; R65.2; T79.4; T81.1; G92.; G93.1; G93.4; G93.5; G93.6; G93.89; G93.9; I60.; F06.8; G13.8; G21.0; G25.7; G25.89; G25.9; G26.; G30.; G31.0; G31.1; G31.83; G31.84; G31.85; G31.89; G31.9; G91.1; G91.2; G91.3; G91.8; G91.9; G94.; I69.010; I69.011; I69.014; I69.015; I69.018; I69.019; I69.110; I69.111; I69.114; I69.115; I69.118; I69.119; I69.210; I69.211; I69.215; I69.218; I69.219; I69.310; I69.311; I69.314; I69.315; I69.318; I69.319; I69.810; I69.811; I69.814; I69.815; I69.818; I69.819; I69.91; R41.1; R41.2; R41.3; R41.81; G20.; G21.1; G21.2; G21.3; G21.4; G21.8; G21.9; M62.5; M62.81; M62.84; M62.89; R53.81; R54.; Z74.0; Z74.01; L89.; L97.; L98.4; G81.; G82.; G83.1; G83.2; G83.3; G83.5; G83.89; G83.9; I69.03; I69.04; I69.05; I69.06; I69.13; I69.14; I69.15; I69.16; I69.23; I69.24; I69.25; I69.26; I69.33; I69.34; I69.35; I69.36; I69.83; I69.84; I69.85; I69.86; I69.93; I69.94; I69.95; I69.96; R29.5  *HCPCS/CPT code:* 97110; 97161; 97162; 97116; 97535; 92507; 97164; 97012; 97112; 97530; 97113; E1390; E1391; E1392; E0431; E0433; E0434; E0435; E0439; E0441; E0442; E0443; A0426; A0427; A0428; A0429; A0999; E1050; E1060; E1070; E1083; E1084; E1085; E1086; E1087; E1088; E1089; E1090; E1091; E1092; E1093; E1100; E1110; E1140; E1150; E1160; E1161; E1170; K0001; K0002; K0003; K0004; K0005; K0006; K0007; K0008; K0009; E0250; E0251; E0255; E0256; E0260; E0261; E0265; E0266; E0270; E0290; E0291; E0292; E0293; E0294; E0295; E0296; E0297; E0301; E0302; E0303; E0304; E0316 |

**References**

1. Larochelle MR, Lodi S, Yan S, Clothier BA, Goldsmith ES, Bohnert ASB. Comparative Effectiveness of Opioid Tapering or Abrupt Discontinuation vs No Dosage Change for Opioid Overdose or Suicide for Patients Receiving Stable Long-term Opioid Therapy. *JAMA Netw Open*. 2022;5(8):e2226523. doi:10.1001/jamanetworkopen.2022.26523

2. Duchesneau ED, Shmuel S, Faurot KR, et al. Translation of a Claims-Based Frailty Index From the *International Classification of Diseases, Ninth Revision, Clinical Modification* to the Tenth Revision. *American Journal of Epidemiology*. 2023;192(12):2085-2093. doi:10.1093/aje/kwad151
